# Supplementary material for: GWAS and Transcriptome Analysis Reveal Key Genes Affecting Root Growth under Low Nitrogen Supply in Maize
Source: Genes (Basel). 2022 Sep 11;13(9):1632. doi: 10.3390/genes13091632 (PMC9498817; doi:10.3390/genes13091632)

Figure S1: Plot of the effect of each principal component.

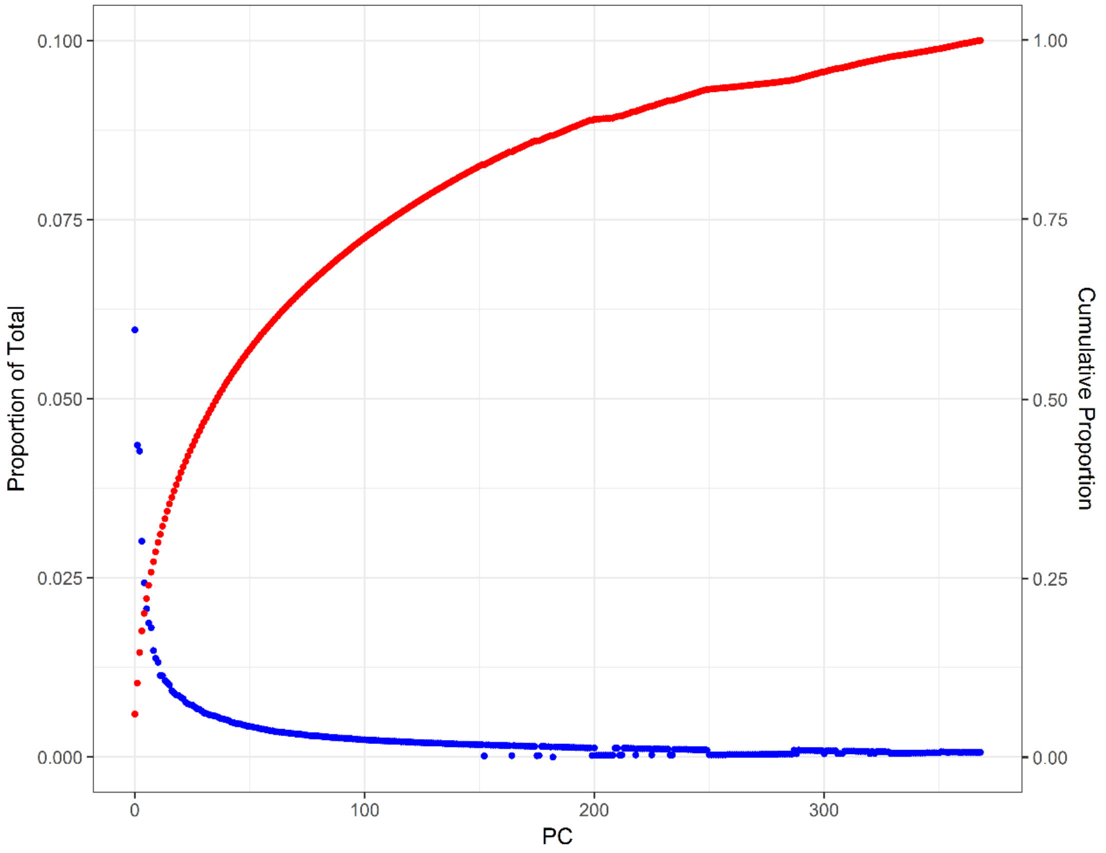

Figure S2: The graph of linkage disequilibrium (LD) decay in all inbred lines.

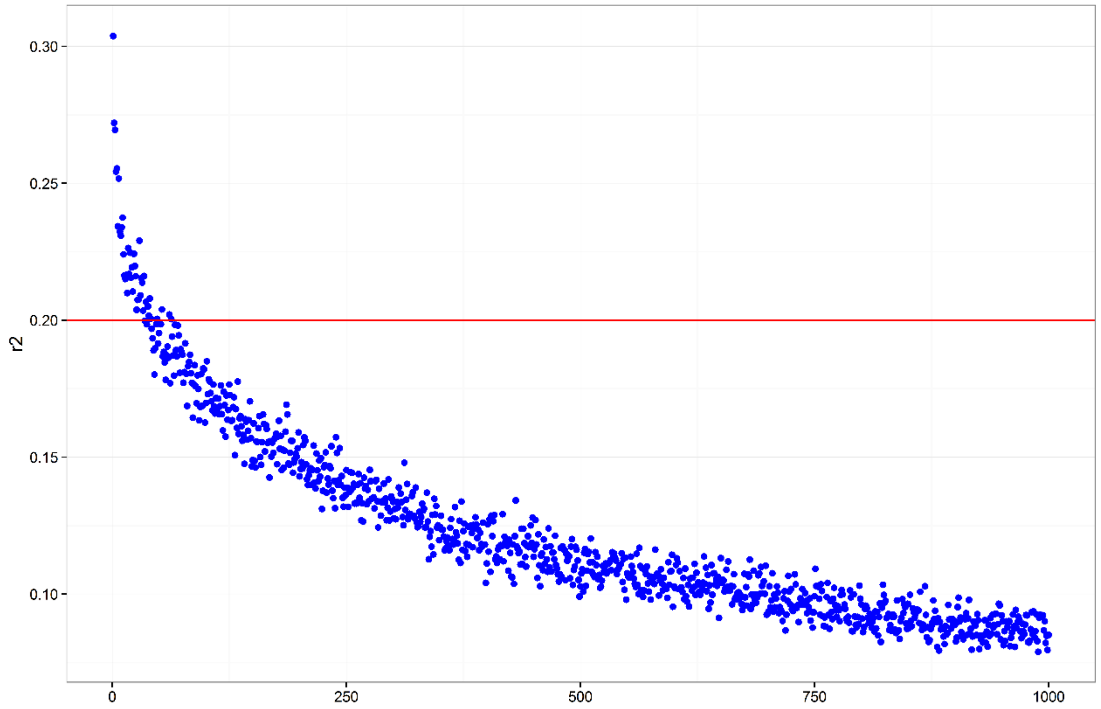

Figure S3: Pearson correlation of the traits under control (CK) and low-N (LN) conditions.

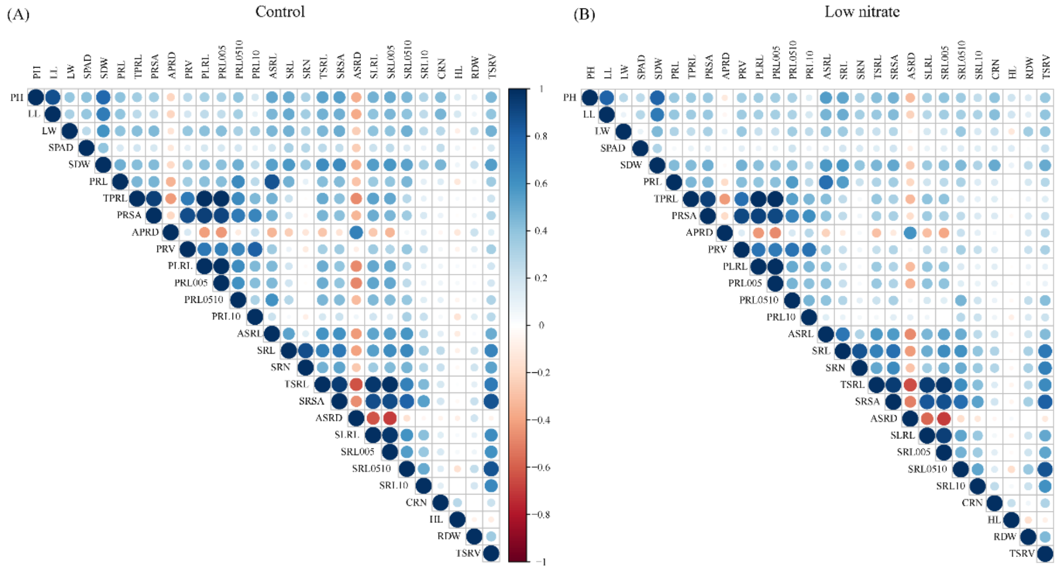

Figure S4: Principal component analysis (PCA) plot showing clustering of traits in 213 maize inbred lines.

(A)

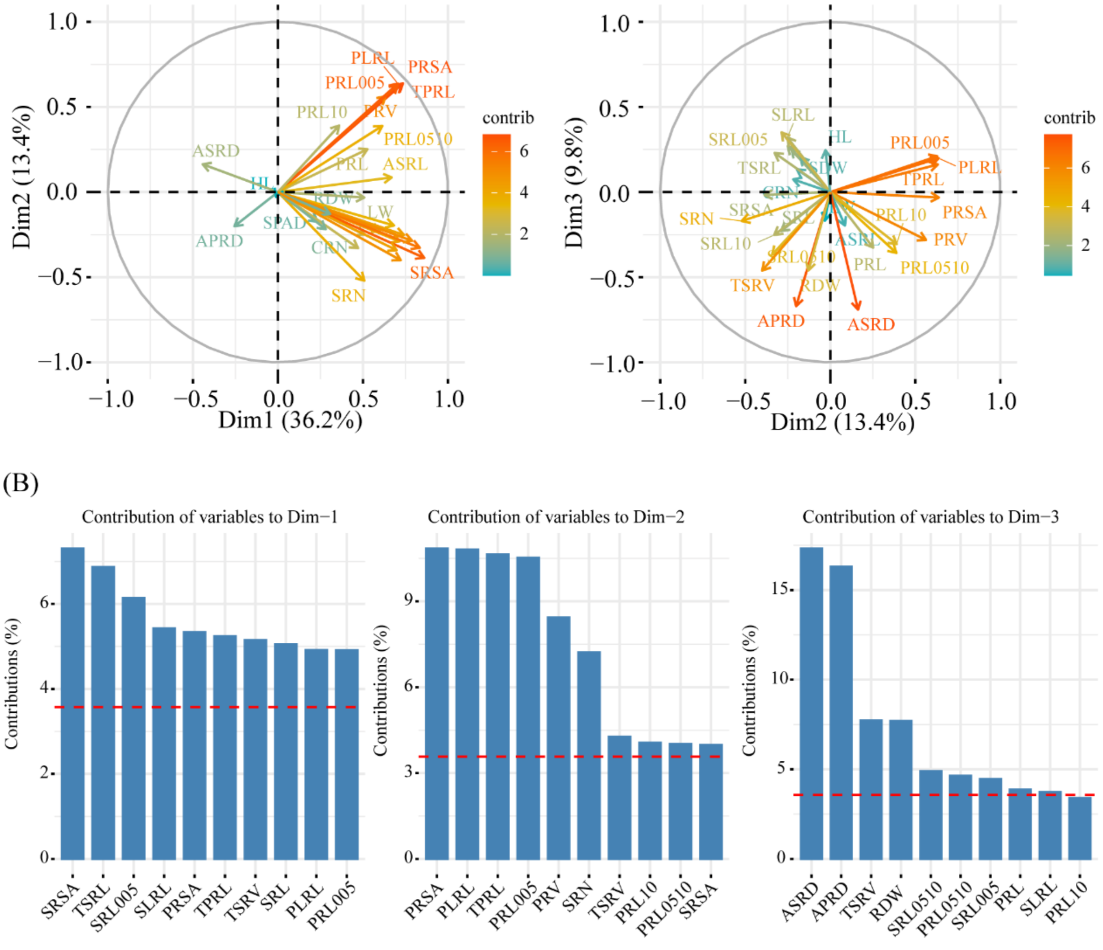

Figure S5: The association networks between SNPs and traits. The white nodes represent traits, and the colored nodes represent SNPs which were significantly associated with corresponding traits. The green, red, and blue edges between traits and SNPs indicated SNPs were identified in control, low nitrate, and the trait plasticity, respectively. LW, leaf width; SPAD; SDW, shoot dry weight; PRL, primary root length; TPRL, total length of primary root; PRSA, primary root surface area; APRD, average diameter of primary root; PLRL, lateral root length of primary root; PRL005, primary root length between 0 and 0.5mm in diameter; PRL10, primary root length greater than 1.0 mm in diameter; ASRL, average length of Seminal roots; SRN, seminal roots numbers; SRSA, seminal root surface area; ASRD, average diameter of seminal root; TSRV, total seminal root volume; SLRL, lateral root length of seminal root; SRL005, seminal root length between 0 and 0.5mm in diameter; SRL0510, seminal root length between 0.5mm and 1mm in diameter; SRL10, seminal root length greater than 1.0 mm in diameter; CRN, crown root number; HL, hypocotyl length; RDW, root dry weight.

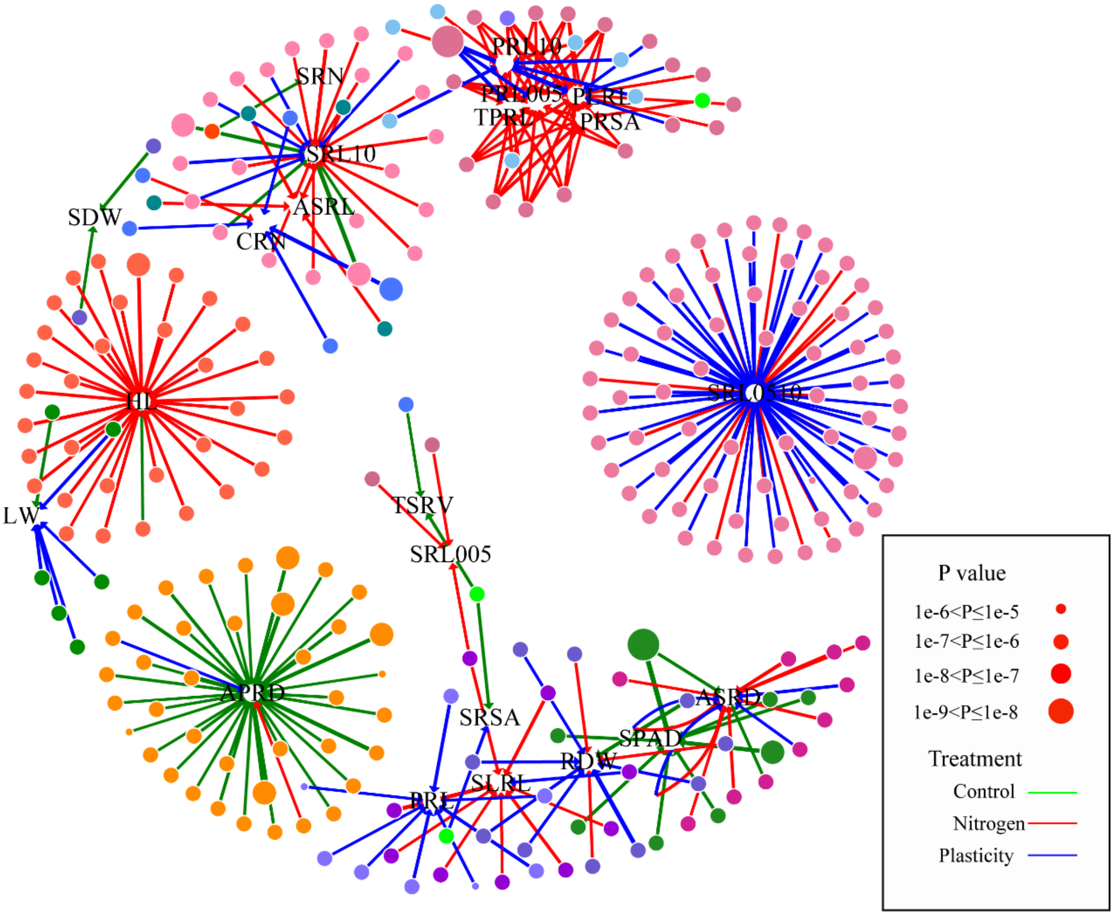

Figure S6: Manhattan plots of associated traits under control (CK) condition.

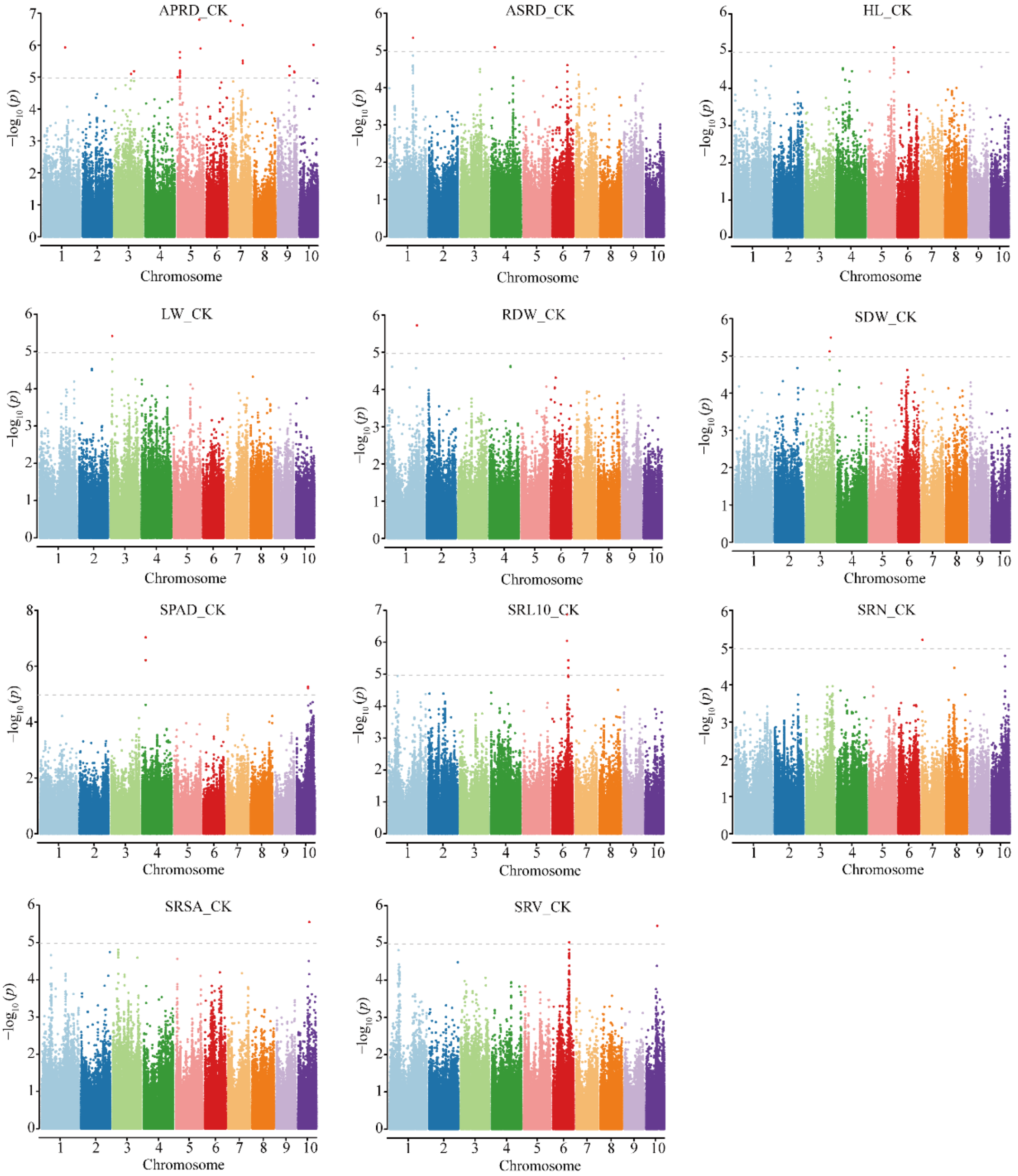

Figure S7: Manhattan plots of N response value which was significantly associated with SNPs.

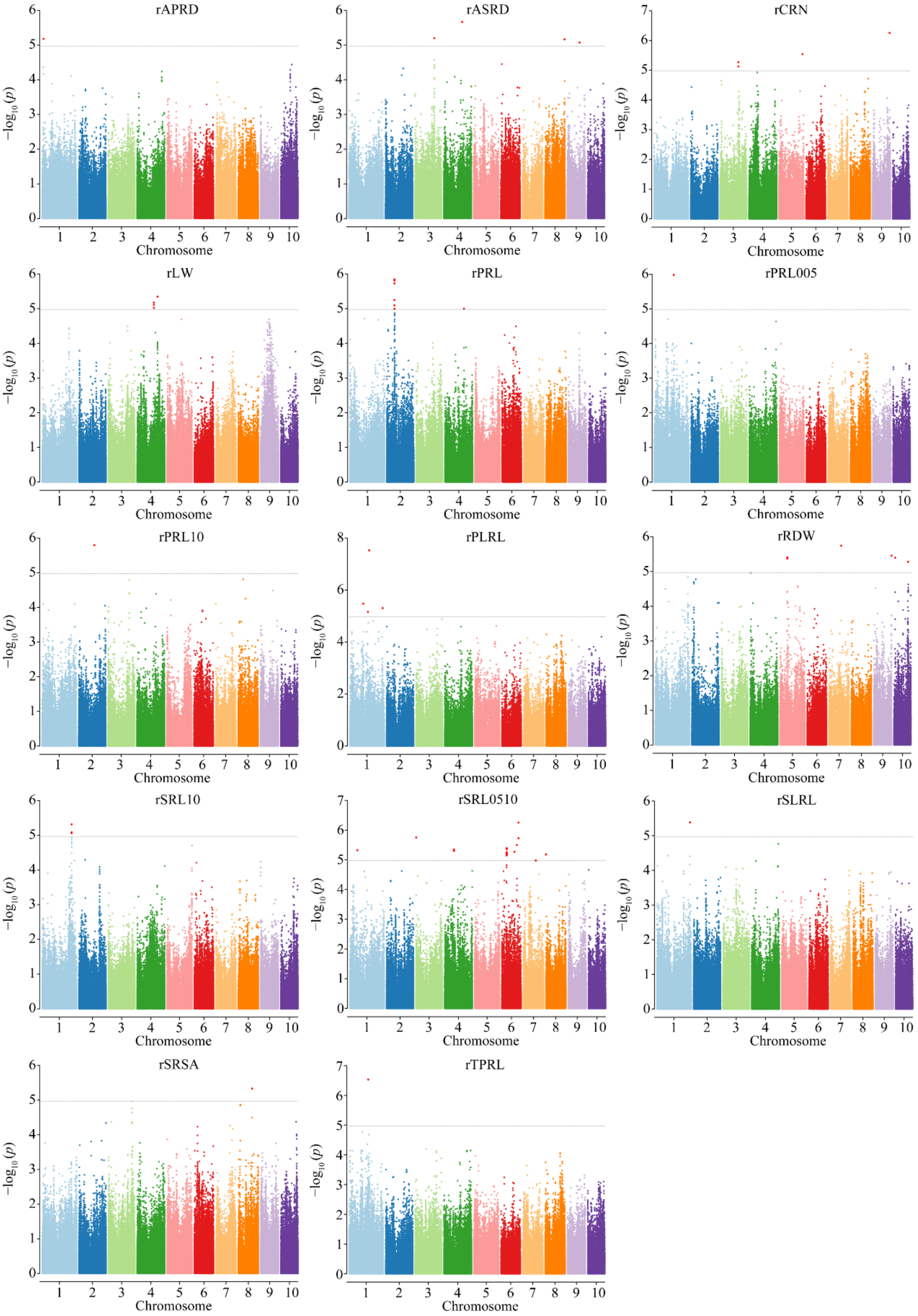

Figure S8: Global gene expression in B73 root under control (CK) and low-N (LN) conditions. (A) Total number of genes expressed in different time point of root development in B73 root under CK and LN conditions. (B) The bar plot shows the fraction of genes expressed at different expressed levels.

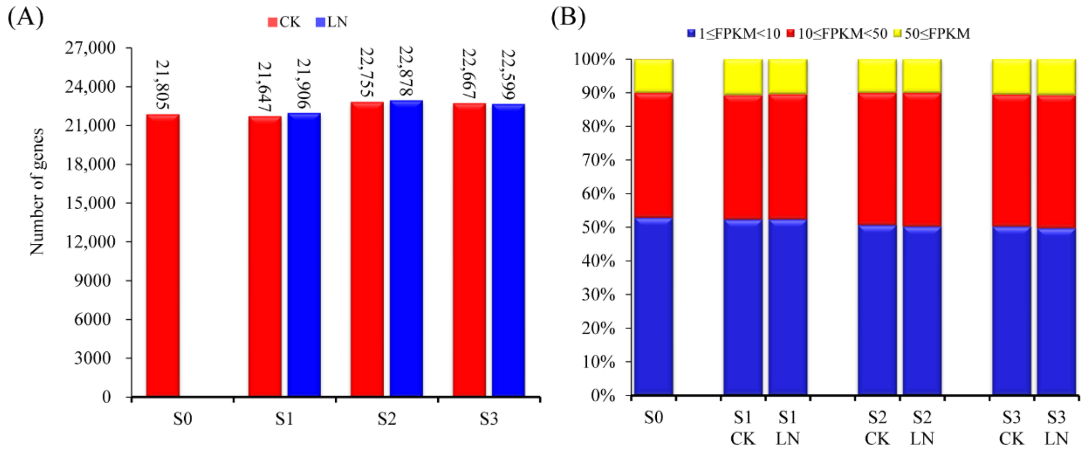

Figure S9: Correlation between expression profiles of seven candidate genes revealed from RNA-seq and RT-qPCR.

Heatmaps showed expression profiles of seven candidate genes (labelled on left side) obtained from RNA-seq (left) and RT-qPCR (right). The values between two heatmaps represent the pearson's correlation (*r*) for each gene between RNA-seq and RT-qPCR. * indicates a statistical significance at $P < 0.05$ level, ** indicates a statistical significance at $P < 0.01$ level.

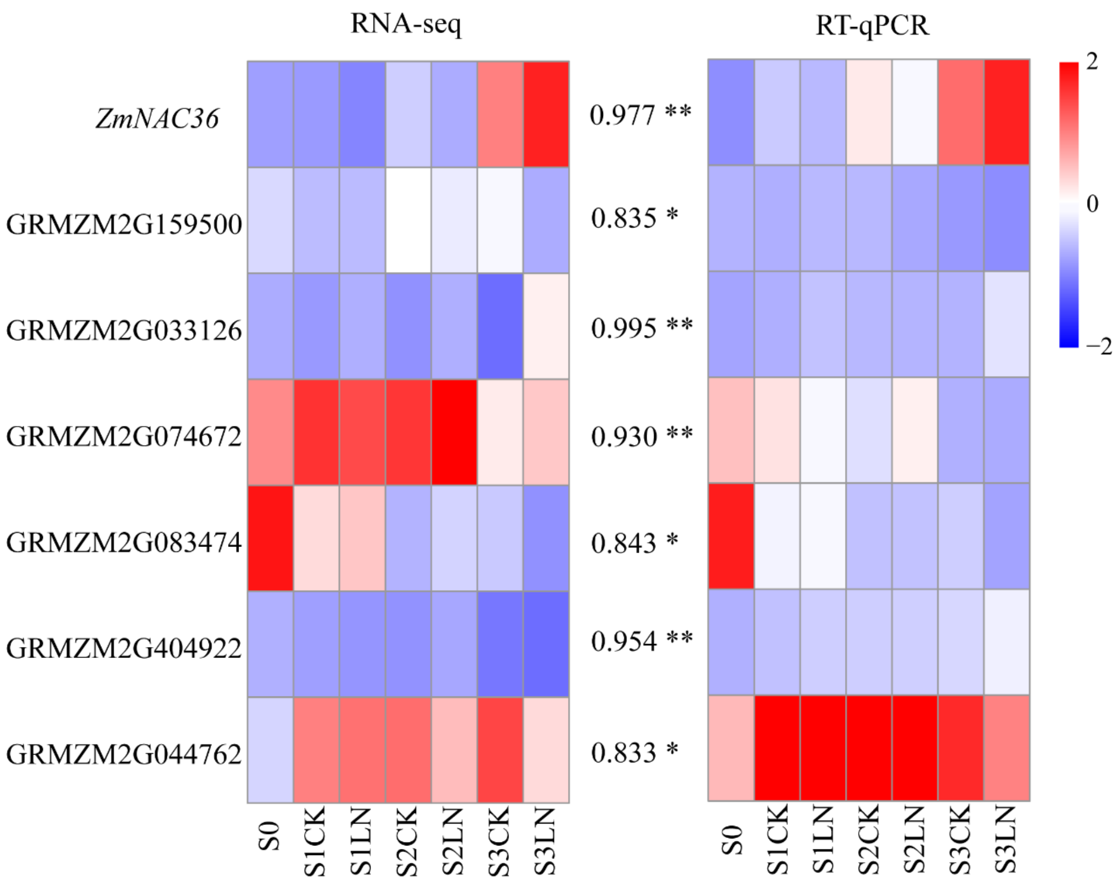

Figure S10: Linkage disequilibrium (LD) heatmap for the significant variants associated with PRL005_LN, PLRL_LN, PRSA_LN, and TPRL_LN.

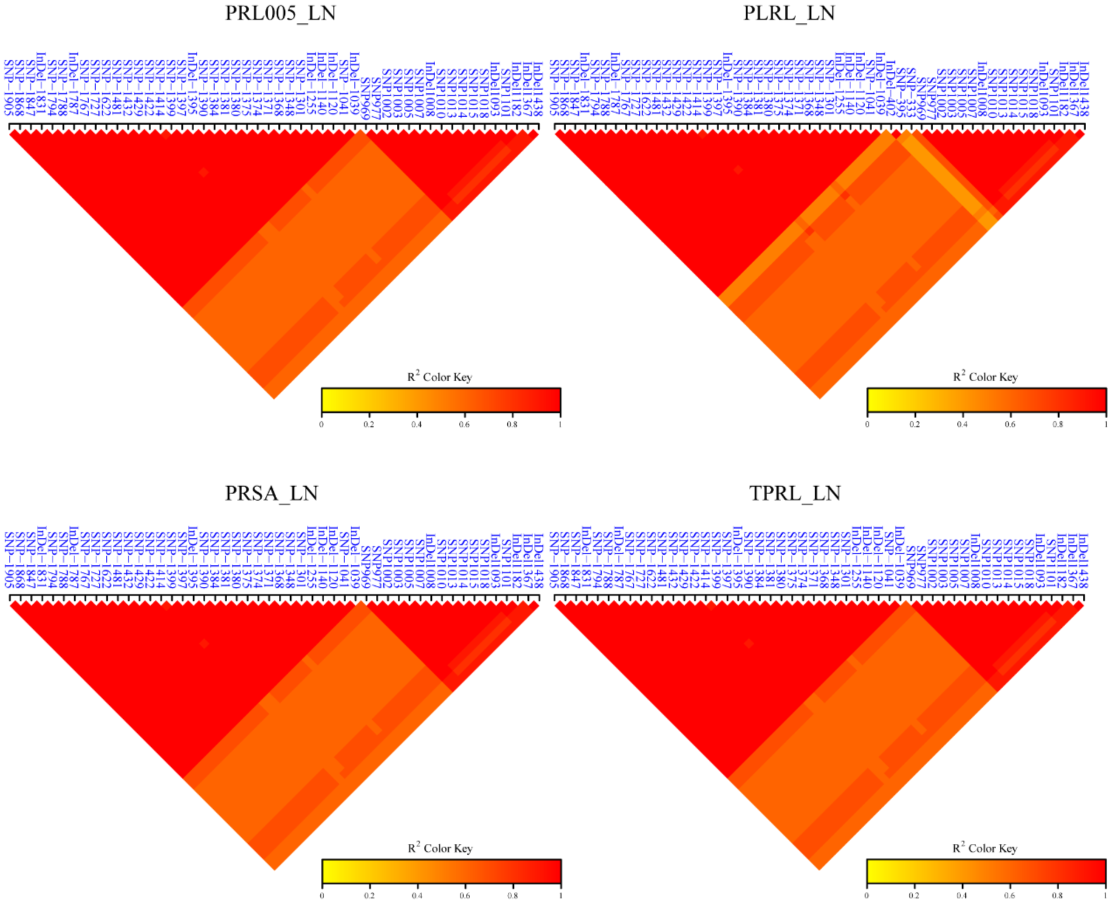

Figure S11: Schematic diagram of the *cis*-regulatory elements of *ZmNAC36* promoter in two haplotypes. The possible *cis*-regulatory elements of *ZmNAC36* promoter were indicated by black boxes. The SNPs and insertions were indicated by red triangles and blue boxes, respectively.

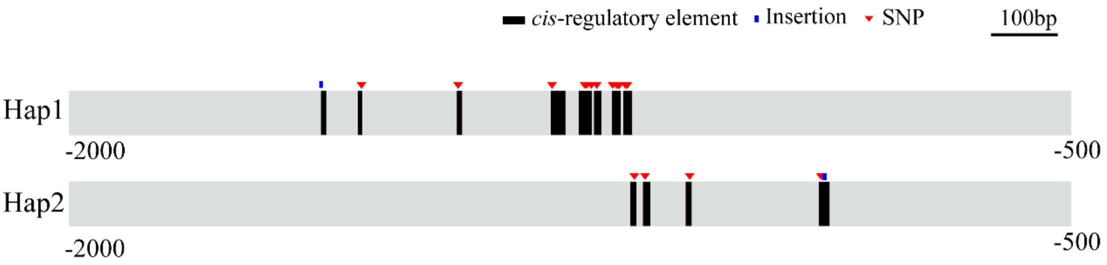

Supplement: Supplementary file 1 [file genes-13-01632-s001.zip › Supplementary Figure S1¿CS11.pdf]
